# Antimicrobial Coatings from Hybrid Nanoparticles of Biocompatible and Antimicrobial Polymers

**DOI:** 10.3390/ijms19102965

**Published:** 2018-09-28

**Authors:** Carolina Nascimento Galvão, Luccas Missfeldt Sanches, Beatriz Ideriha Mathiazzi, Rodrigo Tadeu Ribeiro, Denise Freitas Siqueira Petri, Ana Maria Carmona-Ribeiro

**Affiliations:** 1 Biocolloids Laboratory, Departamento de Bioquímica, Instituto de Química, Universidade de São Paulo, Av. Prof. Lineu Prestes 748, 05508-000 São Paulo, Brazil; carolinagalvao@usp.br (C.N.G.); luccas.sanches@hotmail.com (L.M.S.); bemathi@usp.br (B.I.M.); rodrigo@iq.usp.br (R.T.R.); 2 Departamento de Química Fundamental, Instituto de Química, Universidade de São Paulo, Av. Prof. Lineu Prestes 748, 05508-000 São Paulo, Brazil; dfsp@usp.br

**Keywords:** coatings from nanoparticles, biocompatible polymer, antimicrobial polymer, dynamic light scattering, coatings wettability, microbicidal coatings, bacteria viability, bactericidal coatings, *Escherichia coli*, *Staphylococcus aureus*

## Abstract

Hybrid nanoparticles of poly(methylmethacrylate) synthesized in the presence of poly (diallyldimethyl ammonium) chloride by emulsion polymerization exhibited good colloidal stability, physical properties, and antimicrobial activity but their synthesis yielded poor conversion. Here we create antimicrobial coatings from casting and drying of the nanoparticles dispersions onto model surfaces such as those of silicon wafers, glass coverslips, or polystyrene sheets and optimize conversion using additional stabilizers such as cetyltrimethyl ammonium bromide, dioctadecyldimethyl ammonium bromide, or soybean lecithin during nanoparticles synthesis. Methodology included dynamic light scattering, determination of wettability, ellipsometry of spin-coated films, scanning electron microscopy, and determination of colony forming unities (log CFU/mL) of bacteria after 1 h interaction with the coatings. The additional lipids and surfactants indeed improved nanoparticle synthesis, substantially increasing the conversion rates by stabilizing the monomer droplets in dispersion during the polymerization. The coatings obtained by spin-coating or casting of the nanoparticles dispersions onto silicon wafers were hydrophilic with contact angles increasing with the amount of the cationic polymer in the nanoparticles. Against *Escherichia coli* and *Staphylococcus aureus*, bacteria cell counts were reduced by approximately 7 logs upon interaction with the coatings, revealing their potential for several biotechnological and biomedical applications.

## 1. Introduction

Biomimetic hybrid coatings have often been used as antibacterial materials [1,2,3,4]. For example, silver nanoparticles (NPs) embedded on dextran films or on a lysozyme/dextran network of natural polymers can be grafted onto a variety of surfaces with several biomedical applications possible from coating implants to catheters [5,6,7]. Biocompatible and antimicrobial polymers can be combined to yield a variety of nanostructures, among them, the popular and very useful NPs, which may further form coatings and films [8,9,10]. Antimicrobial polymeric NPs of poly(methylmethacrylate) (PMMA) synthesized in the presence of the cationic antimicrobial polymer poly(diallyldimethyl ammonium) chloride (PDDA) were first obtained in 2015 joining the biocompatible character of PMMA with the microbicide character of the cationic PDDA [11]. PMMA belongs to the Eudragit trademark that includes a diverse range of poly(methacrylate) and polyacrylate-based copolymers which are non-biodegradable, non-absorbable, and nontoxic with several applications in drug delivery [12]. The pharmaceutical applications of polyacrylates for coatings and films were recently and comprehensively reviewed [13].

On the other hand, PDDA was first described as a cationic antimicrobial polymer about 10 years ago, displaying outstanding activity as a microbicide and fungicide [7,14,15,16]. However, the synthesis of hybrid PMMA/PDDA NPs by emulsion polymerization in absence of surfactant yielded low conversion percentiles [11]. This was consistent with previously described and not very successful attempts to polymerize methyl acrylate (MA) or methyl methacrylate (MMA) using large amounts of monomer (>1.9 wt %) in oil-in-water microemulsions for which phase separation during polymerization took place [17,18,19]. The two major steps in emulsion polymerization are nucleation and particle growth. In the presence of surfactant, if the monomer has high affinity for the micelle core, nucleation occurs in the micelles where the monomers are. If the monomer is polar to a certain extent, there will be some affinity for the water phase so that polymerization also occurs in monomer droplets [20,21]. The initiator generates free radicals that react with MMA in the micelles and with MMA inside the droplets in the aqueous phase, yielding oligo radicals that colocalize with the monomers and proceeding with the polymerization. Apparently, the presence of PDDA during NPs synthesis in the absence of surfactant stabilized the smaller droplets of MMA yielding PMMA/PDDA hybrid and very small NPs [11]. Coatings prepared by spin-coating of PMMA and dioctadecyldimethyl ammonium bromide (DODAB) cationic lipid revealed a good compatibility between DODAB and PMMA leading to good antimicrobial activity against bacteria upon contact [22]. The dependence of the antimicrobial activity on the quaternary ammonium compound structure for combinations of PMMA and DODAB, cetyl trimethylammonium bromide (CTAB), or tetra propyl bromide (TPAB) for spin-coated films also yielded interesting results [23]. DODAB remained associated with PMMA films and killed bacteria upon contact, in contrast to CTAB that diffused out of the films killing bacteria in the outer medium [23]. In dispersion, PMMA/DODAB or PMMA/CTAB NPs prepared by emulsion polymerization over a range of high concentration of the quaternary ammonium amphiphiles showed remarkable antimicrobial activity over a range of micromolar concentrations [24].

Here we present some novel antimicrobial coatings based on hybrid NPs of PMMA and PDDA and solve the problem of low conversion during emulsion polymerization for PMMA/PDDA NPs synthesis by adding amphiphiles such as DODAB, CTAB, and lecithin in the reaction mixture. The results revealed remarkable microbicidal activity for the PMMA/PDDA coatings obtained from casting and drying PMMA/PDDA NPs and a substantial increase in conversion due to the presence of the amphiphiles during PMMA/PDDA NPs synthesis.

## 2. Results and Discussion

### 2.1. Physical Properties and Microbicidal Activity of Coatings from PMMA/PDDA Dispersions

The synthesis of PMMA/PDDA NPs, described previously by Sanches et al. [11], yielded monodisperse and cationic NPs in water dispersion named in accordance with MMA and PDDA concentrations used in the particles synthesis. For the dispersions A4, the concentrations used were 0.56 M MMA and 4 mg/mL PDDA; for A5, they were 0.56 M MMA and 5 mg/mL PDDA; and for B4, they were 1.32 M MMA and 4 mg/mL PDDA.

**Figure 1:**
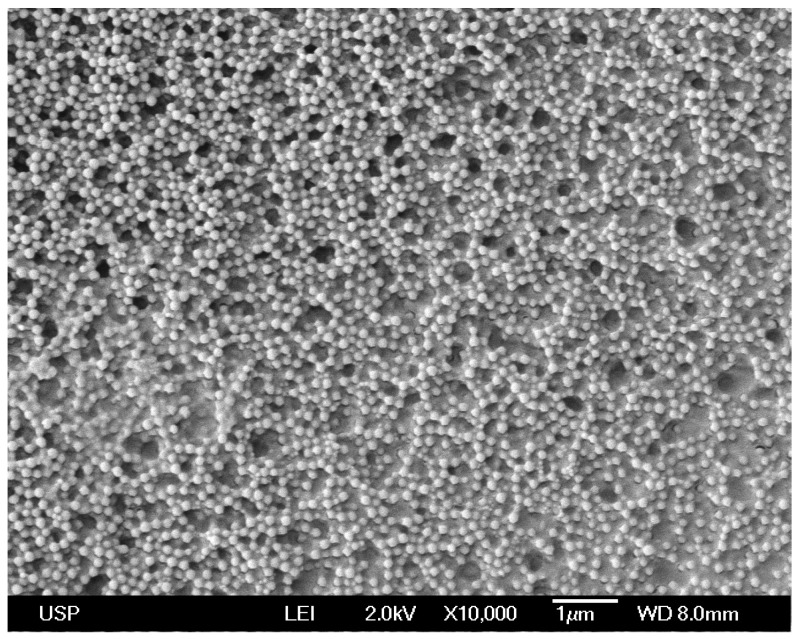
Scanning electron micrograph of the poly(methylmethacrylate) (PMMA)/poly(diallyldimethyl ammonium) chloride (PDDA) coating obtained by casting 0.050 mL of the original A4 nanoparticles dispersion (10 mg/mL) onto silicon wafers.

**Figure 2:**
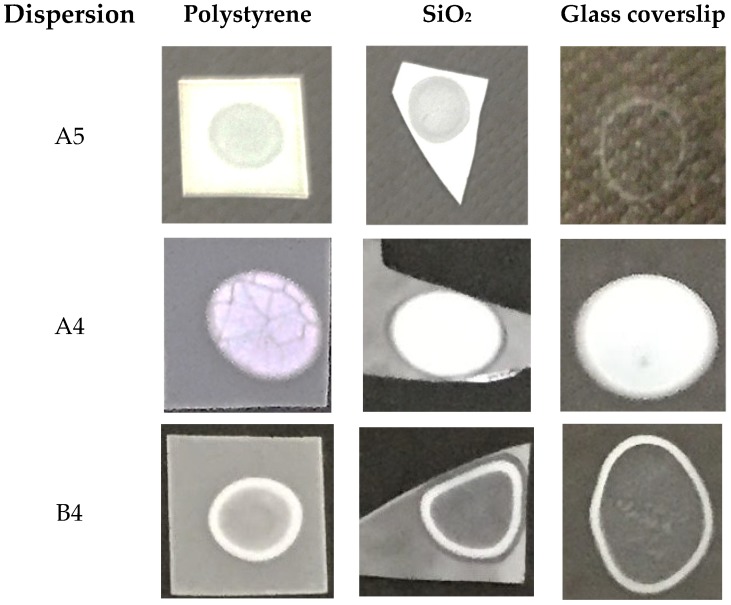
PMMA/PDDA films casted from 50 μL droplets of A5 (4.4 mg/mL), A4 (10 mg/mL), and B4 (5.8 mg/mL) dispersions on polystyrene sheets, silicon wafers, or glass coverslips.

NPs in A4 have a mean diameter of 112 ± 17 nm as determined by Scanning Electron Microscopy (SEM) [11]. Casting and drying the original A4 dispersion on silicon wafers yielded the coating shown on the SEM micrograph (Figure 1), with the macroscopic features for the film seen on Figure 2. The coatings were homogeneous on the hydrophilic surfaces such as the silicon wafers and the glass coverslips. However, cracks and discontinuities were visible for those on the hydrophobic polystyrene substrates (Figure 2). The NPs structure was shown to involve a PMMA core surrounded by a PDDA shell [11] proving that the outer cationic and hydrophilic layer clearly interacted better with the hydrophilic surfaces such as those of the silicon wafer or the glass. The coating adhesion to the hydrophilic and anionic substrates was clearly better for A5 and A4-derived coatings than for those derived from B4 (Figure 2). The reason for this can be related to the higher relative ratio of PDDA to PMMA in A5 and A4-derived coatings than in the B4-derived ones. The interpretation for the ring appearing after casting B4 dispersion was related to the coffee-ring effect; such ring deposition occurs when liquid evaporation from the edge is replenished by liquid from the interior so that the resulting outward flow can carry most of the dispersed material to the edge [25]. This took place for the most hydrophobic NPs, namely, those with the lowest PDDA:PMMA molar ratios represented by the B4 dispersion. Similar ring deposition pattern was also observed for hydrophobic polystyrene particles deposited on glass from a water droplet and explained from the coffee ring effect [26]. The crack patterns visible for A4-derived coating on the polystyrene substrate were radial and similar to the ones previously described in the literature for similar systems [27]. The poor adhesion of the hydrophilic NPs of the A4 derived-coating to the hydrophobic polystyrene sheet might also have contributed to cracks in the coating (Figure 2).

PMMA/PDDA coatings on silicon wafers are derived from two different procedures: (1) spin-coating of lyophilized A5 in 1:1 dichloromethane: ethanol; (2) casting of A5, A4, or B4 dispersions of NPs followed by drying under vacuum.

**Figure 3:**
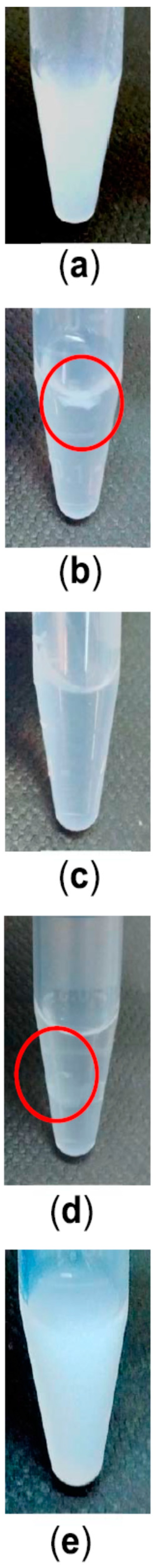
Checking solvent mixtures for the solubilization of lyophilized PMMA/PDDA A5 dispersion (1 mL) aiming at the preparation of coatings by spin-coating. Solvents were dichloromethane (**a**); 75% dichloromethane: 25% ethanol (**b**); 50% dichloromethane: 50% ethanol (**c**); 25% dichloromethane: 75% ethanol (**d**); and ethanol (**e**). The red circles emphasize the fact that some insoluble polymer still remained in the solvents mixture.

Spin-coating allows for the preparation of lipid [28,29,30] or polymer films [22] on very smooth surfaces such as those of the silicon wafers. In the present case, the composition of a hydrophobic polymer, such as PMMA, and a hydrophilic one, such as PDDA, required a special combination of solvents in order to obtain solubilization of both in the solvents mixture. Figure 3a–e shows the evaluation of solubilization of both polymers from the lyophilized A5 dispersion in ethanol (E):dichloromethane (D) over a range of E:D proportions. The complete solubilization only took place at 50:50% E:D. This allowed obtaining the coatings of PMMA: PDDA onto silicon wafers for evaluation of thickness, refractive index and contact angles (Table 1). These characteristics of the hybrid films compared to those of pure PMMA coatings revealed similar thicknesses and refractive indices but higher wettability for the hybrid coatings than those determined for the pure PMMA film (Table 1).

For coatings obtained by casting the PMMA/PDDA dispersions onto the silicon wafers, there was a consistent decrease of the contact angle upon increasing the PDDA relative amount in the dispersions from 35 ± 6 to 9 ± 2 degrees (Table 1). Coatings obtained by casting the dispersions yielded lower contact angles than those obtained by spin-coating, reconfirming that the hydrophilic PDDA immobilized as an outer layer of the PMMA/PDDA nanoparticle imparted a more hydrophilic character to the film surface than the one of the spin-coated PMMA/PDDA (Table 1).

The antimicrobial activity of the hybrid PMMA/PDDA coatings derived from A5, A4, and B4 casted onto glass coverslips revealed a remarkable microbicidal effect against *Escherichia coli* and *Staphylococcus aureus* (Table 2). In this case, the real potency of the coatings was established over orders of magnitude by determining bacteria viability from the log of CFU/mL. Bacteria viability decreased by 10^7^–10^8^ colony forming units (CFU) upon interaction with the coatings for 1 h (Table 2).

### 2.2. Optimization of Nanoparticles Synthesis and Conversion Percentiles

**Figure 4:**
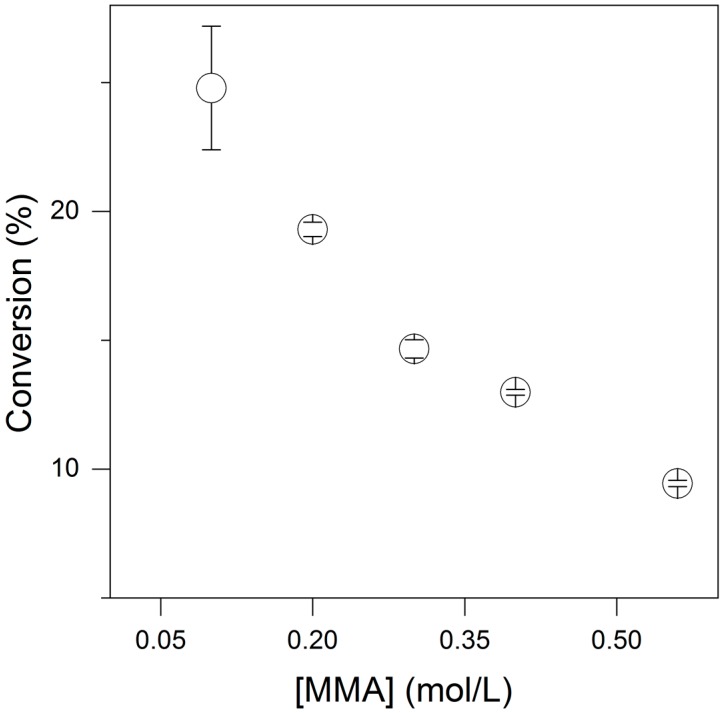
The effect of methyl methacrylate (MMA) concentration on conversion (%) of MMA into PMMA in the presence of PDDA (5 mg/mL) and AIBN (0.36 mg/mL) as initiator. The nanoparticles synthesis proceeded for 2 h at 70 to 80 °C and was followed by extensive dialysis before performing the dispersions characterization by dynamic light-scattering shown on Table 3.

The synthesis of PMMA/PDDA NPs as dispersion A5 in absence of surfactants displayed low monomer-into-polymer conversion since only approximately 10% of the monomer mass added was converted into polymer [11]. In order to improve conversion percentiles, the effect of monomer concentration on conversion was determined (Figure 4; Table 3). At 5 mg/mL PDDA, decreasing the methylmethacrylate (MMA) concentration [MMA] improved conversion, and possible reasons for this would be the relative increase in PDDA capable of stabilizing the droplet/water interface and the increased average distance between MMA droplets reducing coalescence. One should note that NPs size could also be reduced by decreasing [MMA] meaning that polymerization from smaller droplets yielded smaller NPs. At this point, stabilizing the droplet/water interface seemed crucial for improving conversion. Therefore, CTAB, DODAB, and lecithin were introduced in the reaction mixture for further stabilization of the monomer droplets.

In fact, all amphiphiles employed improved conversion (Table 4). The most efficacious amphiphile was CTAB, followed by DODAB and lecithin. Since lecithin corresponds to a mixture of lipids and fatty acids with a net negative charge [31,32], at 2 mM lecithin, the NPs became negatively charged; all other NPs exhibited high and positive zeta-potentials (Table 4). In the presence of two stabilizers (amphiphile and PDDA), conversion was substantially increased in comparison to the one in the presence of a single stabilizer. Another interesting observation refers to the lower zeta-potential for PMMA/CTAB in comparison to the one for PMMA/DODAB; this is consistent with the reported immobilization of DODAB in the PMMA polymeric matrix which is absent for CTAB, since CTAB was reported to be more mobile than DODAB easily diffusing to the outer medium from PMMA films [22,23]. In summary, although amphiphiles indeed improved conversion, PDDA as a second stabilizer possibly provided an additional stabilizing factor, which was the electrosteric repulsion between the MMA droplets during NP synthesis. This also represented an important stabilizing factor for the final polymeric NPs.

**Figure 5:**
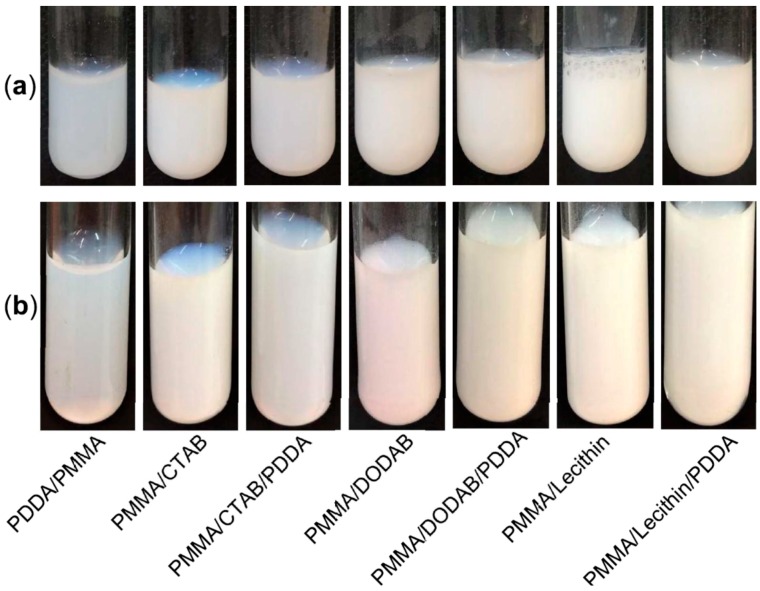
(**a**) Photos of dispersions just after synthesis and dialysis; (**b**) photos of the same dispersions approximately 4 months later. Details on composition and physical properties of the dispersions just after synthesis are on Table 5.

Figure 5 and Table 5 show the remarkable colloidal stability of the NPs characterized by the physical properties on Table 4. The photos taken one day and 4 months after synthesis revealed very similar macroscopic features and absence of precipitates. The analysis of sizes, polydispersities, and zeta-potentials also revealed maintenance of these physical properties of the NPs over time (Table 5).

As compared to other similar systems in the literature, the present NPs use the self-assembly of biocompatible PMMA and the antimicrobial polymer PDDA instead of synthesizing block copolymers incorporating both functions. For example, glycosylated block copolymers were used as surfactants in butyl methacrylate emulsion polymerization [33]. However, the antimicrobial activity was not as high as the one obtained for the coatings described in this work (Table 2). The higher hydrophobicity inherent to the two methyl groups on the quaternary nitrogen of the PDDA molecule, as compared to the cationic glycosylated moieties, was an advantage for efficient microbicide activity. Indeed, several derivatives of PDDA evaluated for their antimicrobial activity revealed that these cationic polymers exhibit the highest activity when their chemical structure bears high frequency of hydrophobic methyl moieties [11,34]. The hydrophilic character of cationic antimicrobial polymers does not contribute to improvement of the antimicrobial action, although the NPs synthesis certainly benefits from their use as surfactants.

A major drawback of PMMA/PDDA NPs synthesis was the low conversion due to the relatively poor function of PDDA at the interface of MMA droplets and the surrounding water medium during NP synthesis (Figure 4). In this work, we solved this problem by adding amphiphiles such as CTAB, DODAB, and lecithin as surfactants active as stabilizers during the NPs synthesis. In addition, we must recognize the excellent perspective of these ternary systems as antimicrobials since PDDA, DODAB, and CTAB have already being described in separate as good antimicrobial agents [3,8,10,14,24,35,36,37]. The antimicrobial properties of these ternary systems both as latexes dispersions in water and as coatings still have to be determined.

## 3. Materials and Methods

### 3.1. Materials

MMA, PDDA, azobisisobutyronitrile (AIBN), NaCl, CTAB, DODAB, soybean lecithin, chloroform, ethanol, dichloromethane, and Mueller–Hinton agar (MHA) were purchased from Sigma-Aldrich (Darmstadt, Germany) and used without further purification. The composition of soybean lecithin includes several fatty acids and phospholipids [31,32]. Silicon (100) wafers were from Silicon Quest (Santa Clara, CA, USA) with a native oxide layer approximately 2 nm thick and used as substrates for casting the dispersions. These Si wafers with a native SiO_2_ layer were cut into small pieces of ca 1 cm^2^, cleaned with acetone, and dried under a N_2_ stream; they are smooth substrates for the coatings. The syntheses in 1 mM NaCl solution prepared with Milli-Q water yielded NPs dispersions by emulsion polymerization that underwent dialysis for purification using a cellulose acetate dialysis bag with molecular weight cut-off around 12,400 g/mol. All other reagents were analytical grade and used without further purification.

### 3.2. Preparation of NPs by Emulsion Polymerization

A variety of hybrid and polymeric NPs were obtained by polymerization of MMA at 70 to 80 °C for 1 h using 10 mL of aqueous solutions of NaCl 1 mM and PDDA and/or CTAB, DODAB, or lecithin in accordance with compositions shown in Table 6 [11]. Briefly, a weak flux of nitrogen was applied to the solution during a few minutes before adding 3.6 mg of AIBN initiator and MMA. For dispersions containing surfactants or lipids, DODAB or lecithin were previously dissolved in chloroform in order to prepare lipid films under a nitrogen flux to evaporate the chloroform solvent [38,39]. Ten milliliters of the NaCl 1 mM solution was then added to the dried lipid films before proceeding with NP synthesis. In the case of CTAB, the required amount of CTAB in the NPs dispersion was directly added to the 1 mM NaCl solution before starting the NPs synthesis. The NP dispersions obtained were further purified by dialysis against Milli-Q water until water conductivity reached 5 µS/cm.

### 3.3. Determination of Zeta-Average Diameter (Dz), Polydispersity (P), Zeta-Potential (ζ), and Colloidal Stability of NPs Dispersions

Size distributions, Dz, ζ, and P were obtained by dynamic light-scattering (DLS) using a Zeta Plus Zeta Potential Analyzer (Brookhaven Instruments Corporation, Holtsville, NY, USA) equipped with a laser of 677 nm with measurements at 90°. P of the dispersions was determined by DLS following well defined mathematic equation [40]. Dz values were obtained from the log normal distribution of the light-scattered intensity curve against the diameter. ζ values were determined from the electrophoretic mobility (μ) and Smoluchowski equation ζ = μη/ε, where η and ε are the viscosity and the dielectric constant of the medium, respectively. Samples were diluted 1:30 with a 1 mM NaCl water solution for performing the measurements at (25 ± 1) °C.

The colloidal stability of the dispersions was followed by two procedures: (1) from photographs of the dispersions; (2) from the physical properties (Dz, P, and ζ), both procedures performed at days one and 120.

### 3.4. Preparation of Coatings from the NPs Dispersions by Spin-coating or Casting

For preparing spin-coated films, 1 mL of the A5 dispersion was lyophilized and a 10 mg/mL solution in the solvents mixture (1:1 dichloromethane: ethanol) was prepared; 0.1 mL of this solution was then spin-coated onto silicon wafers using a Headway PWM32-PS-R790 spinner (Garland, TX, USA), operated at 3000 rpm during 40 s, at (24 ± 1) °C, and (50 ± 5)% of relative humidity. Thereafter, the film was characterized by ellipsometry [41] which allowed us to obtain the thickness and refractive index of the film independently [22]. 

Films prepared by casting employed 0.05 mL of A5, A4, or B4 original dispersions casted onto three different surfaces: polystyrene, silicon wafers, or glass coverslips. After drying overnight under vacuum the films were photographed, observed by SEM, characterized regarding their wettability, and used for determining antimicrobial activity.

### 3.5. Physical Characterization of Coatings by SEM, Macroscopic Features from Photographs and Contact Angle Determinations

SEM for the coatings employed Jeol JSM-7401F equipment (JEOL Ltd., Akishima, Tokyo, Japan). In short, 2 µL of A5 dispersion on silicon wafers dried in a desiccator before coverage with a thin gold layer as required for contrast and visualization by SEM.

Coatings from A5, A4, or B4 onto different substrates (polystyrene sheet, silicon wafers, and glass coverslip) were obtained by casting 50 µL onto the substrates and allowing the material to dry overnight in a desiccator before taking pictures or determining wettability by using a home built apparatus, as previously described [29,30]. Photos of sessile water droplets of 10 µL allowed for the determining of the advancing contact angle (Ө_A_) over the first 5 min after depositing the droplet on the films. Each determination was taken as a mean ± the standard deviation of at least 4 measurements.

### 3.6. Microorganisms Growth and Determination of Cell Viability in the Presence of the Coatings

*E. coli* ATCC (American Type Culture Collection) 25322 and *S. aureus* ATCC 29213 growths were purchased from previously frozen stocks and kept at −20 °C in appropriate storage medium. The bacterial strains plated onto MHA were incubated at 37 °C/18–24 h before transferring some isolated colonies to an isotonic 0.264 M d-glucose solution and adjusting turbidity to 0.5 of the McFarland scale [42]. The 0.264 M d-glucose solution was used instead of any culture medium because cationic molecules are inactivated by the relatively high ionic strength or negatively charged molecules such as amino acids and polysaccharides. For determination of cell viability after interaction with the PMMA/PDDA NPs coatings, final bacteria cell concentrations in the suspensions were around 10^8^ CFU/mL. 

Sixty microliters of the bacterial suspensions were deposited on the coatings (obtained by casting of A5, A4, or B4 dispersions onto glass coverslips) and left in a water-vapor-saturated chamber for 1 h to prevent water evaporation from the droplet. Thereafter, the glass coverslips were transferred to 10 mL of 0.264 M d-glucose isotonic solution in Falcon tubes and vigorously stirred by vortexing before withdrawing 0.1 mL aliquots and preparing their 1:10 and 1:100 dilutions for plating on MHA plates, incubating the plates (37 °C/24 h), and reading the CFU. These readings were converted into CFU/mL and log (CFU/mL). When no counting was obtained, since the log function does not exist for zero, the CFU/mL counting was taken as 1 so that log CFU/mL could be taken as zero. Controls were bare glass coverslips.

## 4. Conclusions

PMMA/PDDA nanoparticles coated three different substrates by two different procedures: (1) spin-coating; (2) casting followed by drying of the casted dispersions. Macroscopically homogeneous films without cracks coated the hydrophilic substrates such as silicon wafers or glass coverslips. On hydrophobic substrates such as polystyrene surfaces, the coatings showed cracks after drying. The most homogeneous coatings occurred at the highest relative contents of PDDA:PMMA. Upon lowering PDDA contents in the NPs, the NPs accumulated at the periphery of the droplets casted on the substrates. This was due to the coffee ring effect, since the more hydrophobic NPs followed the capillary flow to the periphery of the droplet. The contact angles for the coatings showed a clear dependence of wettability on the PDDA content of the NPs. The higher the PDDA content, the lower the contact angle and the better the adhesion to oppositely charged hydrophilic substrates. Comparing films obtained by spin-coating with those obtained by casting of the NPs onto the substrates showed that spin-coated coatings had larger contact angles than coatings obtained by casting, suggesting that some PDDA molecules might have migrated to the silicon wafer–water interface hiding from the film surface and therefore becoming somewhat unavailable to kill bacteria at the film surface. There was a remarkable microbicide activity due to 0.8–1.0 mg of PDDA distributed in the coatings: after 1 h interaction with bacteria, their viability decreased by approximately 7 to 8 logs as tested against *E. coli* or *S. aureus* cells. This was possibly due to the more hydrophobic nature of PDDA in comparison with other hydrophilic cationic polymers. 

CTAB, DODAB, or lecithin as additional stabilizers for the PMMA/PDDA NPs synthesis substantially improved conversion of MMA into PMMA. These ternary systems were stable and maintained their macroscopic and microscopic physical characteristics with time (checked for 4 months). The use of these ternary systems as microbicides still needs systematic evaluation.

## Figures and Tables

**Table 1:** Physical properties of PMMA/PDDA coatings on silicon wafers. The procedures for coating were: (1) spin-coating of lyophilized A5 in 1: 1 dichloromethane: ethanol; (2) casting of A5, A4, or B4 NPs dispersions followed by drying under vacuum.

Materials	Procedure	Composition	Thickness/nm	Refractive Index	Contact Angle Ө_A_/degrees
PMMA/PDDA	Spin-coating	Lyophilized A5	94 ± 3	1.495 ± 0.004	15 ± 1
PMMA/PDDA	Casting	A5	-	-	9 ± 2
PMMA/PDDA	Casting	A4	-	-	19 ± 2
PMMA/PDDA	Casting	B4	-	-	35 ± 6
PMMA ^1^	Spin-coating	PMMA	91 ± 1	1.499 ± 0.004	76 ± 5

^1^ Data from reference [22].

**Table 2:** Microbicidal activity of PMMA/PDDA coatings obtained from casting and drying under vacuum A5, A4, or B4 dispersions (0.2 mL) onto glass coverslips. Since 4 or 5 mg/mL of PDDA were the concentrations used for particle synthesis, in 0.2 mL of each dispersion used for the coatings there will be 0.8 to 1.0 mg of PDDA acting against the bacteria.

Dispersion Used for Coating	Microorganism	Initial Cell Viability/log (CFU/mL)	Final Cell Viability/log (CFU/mL)
A5	*E. coli*	7.2	0
A5	*S. aureus*	7.9	0
A4	*E. coli*	7.1	0
A4	*S. aureus*	8.2	0
B4	*E. coli*	7.1	0
B4	*S. aureus*	8.2	0

**Table 3:** Effect of MMA concentration on physical properties of the PMMA/PDDA dispersions obtained by emulsion polymerization of MMA at 5 mg/mL PDDA in 1 mM NaCl using 0.36 mg/mL of AIBN.

[MMA] (M)	Dz (nm)	P	ζ (mV)	Solid Contents (mg/mL)
0.10	153 ± 1	0.04 ± 0.01	+46 ± 5	3.1 ± 0.3
0.20	188 ± 1	0.05 ± 0.01	+52 ± 3	3.4 ± 0.1
0.30	153 ± 1	0.04 ± 0.02	+47 ± 3	4.1 ± 0.1
0.40	244 ± 1	0.02 ± 0.00	+51 ± 2	5.8 ± 0.1
0.56	213 ± 3	0.03 ± 0.02	+54 ± 2	3.8 ± 0.1

**Table 4:** The effect of PDDA, surfactants, and lipids on NPs size (Dz), polydispersity (P), and zeta-potential (ζ) on the stabilization of MMA droplets in water and the improvement of solid contents and conversion percentiles for NPs synthesis.

Dispersion *	Dz (nm)	P	m(V)	Solids (mg/mL)	Conversion (%)
PMMA/PDDA	226 ± 3	0.01 ± 0.01	+51 ± 1	6 ± 1	11 ± 1
PMMA/CTAB	97 ± 0	0.05 ± 0.01	+25 ± 1	38 ± 1	66 ± 1
PMMA/CTAB/PDDA	91 ± 0	0.04 ± 0.01	+47 ± 3	26 ± 1	79 ± 1
PMMA/DODAB	177 ± 1	0.07 ± 0.01	+65 ± 1	16 ± 1	28 ± 1
PMMA/DODAB/PDDA	229 ± 2	0.03 ± 0.02	+45 ± 3	17 ± 1	47 ± 1
PMMA/Lecithin	178 ± 1	0.10 ± 0.01	-27 ± 2	13 ± 1	23 ± 1
PMMA/Lecithin/PDDA	233 ± 1	0.04 ± 0.02	+54 ± 1	8 ± 1	24 ± 1

* Concentrations used for NPs synthesis were: [MMA] = 0.56 M; [PDDA] = 5 mg/ mL; [CTAB] = [DODAB] = [Lecithin] = 2 mM.

**Table 5:** The colloidal stability of NPs dispersions from sizing (Dz), polydispersity (P), and zeta-potential (ζ) for dispersions aged 1 and 120 days after synthesis.

Dispersion *	Dz /nm		P		ζ/mV	
	Day 1	Day 120	Day 1	Day 120	Day 1	Day 120
PMMA/PDDA	226 ± 3	211 ± 3	0.01 ± 0.01	0.05 ± 0.01	+51 ± 1	+55 ± 1
PMMA/CTAB	97 ± 0	95 ± 0	0.05 ± 0.01	0.08 ± 0.01	+25 ± 1	+26 ± 1
PMMA/CTAB/PDDA	91 ± 0	90 ± 1	0.04 ± 0.01	0.04 ± 0.01	+47 ± 3	+50 ± 2
PMMA/DODAB	177 ± 1	176 ± 1	0.07 ± 0.01	0.09 ± 0.02	+65 ± 1	+50 ± 1
PMMA/DODAB/PDDA	229 ± 2	226 ± 1	0.03 ± 0.02	0.04 ± 0.02	+45 ± 3	+54 ± 1
PMMA/Lecithin	178 ± 1	176 ± 1	0.10 ± 0.01	0.16 ± 0.02	⁻27 ± 2	⁻21 ± 1
PMMA/Lecithin/PDDA	233 ± 1	217 ± 2	0.04 ± 0.02	0.03 ± 0.02	+54 ± 1	+55 ± 1

* Concentrations used for NPs synthesis were: [MMA] = 0.56 M; [PDDA] = 5 mg/ mL; [CTAB] = [DODAB] = [Lecithin] = 2 mM.

**Table 6:** Concentrations of MMA, PDDA, cetyl trimethylammonium bromide (CTAB), dioctadecyldimethyl ammonium bromide (DODAB), and/or lecithin used to synthesize hybrid NPs by emulsion polymerization.

Dispersion	[MMA] (M)	[PDDA] (mg/mL)	[CTAB] (mM)	[DODAB] (mM)	[Lecithin] (mM)
A5	0.56	5	-	-	-
A4	0.56	4	-	-	-
B4	1.32	4	-	-	-
PMMA/CTAB	0.56	-	2	-	-
PMMA/CTAB/PDDA	0.56	5	2	-	-
PMMA/DODAB	0.56	-	-	2	-
PMMA/DODAB/PDDA	0.56	5	-	2	-
PMMA/lecithin	0.56	-	-	-	2
PMMA/lecithin/PDDA	0.56	5	-	-	2

## Abbreviations

Ө_A_	Advancing Contact Angle
ATCC	American Type Culture Collection
AIBN	Azobisisobutyronitrile
CTAB	Cetyltrimethylammonium Bromide
CFU	Colony Forming Unities
D	Dichloromethane
ε	Dielectric Constant of the Medium
DODAB	Dioctadecyldimethylammonium Bromide
DLS	Dynamic Light Scattering
μ	Electrophoretic Mobility
E	Ethanol
MA	Methyl acrylate
MMA	Methyl methacrylate
MHA	Mueller-Hinton Agar
NPs	Nanoparticles
P	Polydispersity
PMMA	Poly(methyl methacrylate)
PDDA	Poly(diallyldimethylammonium) chloride
SEM	Scanning Electron Microscopy
η	Viscosity of the Medium
Dz	Zeta-average Diameter
ζ	Zeta-potential
